# Dr. Walter Robarts Gillette

**Published:** 1908-12

**Authors:** 


Dr. Walter Robarts Gillette, of New York, died at the Roosevelt Hospital, November 7, 1908, after an operation for cancer of the intestines, aged 68 years. He was a member of the New York Academy of Medicine, of the Medical Society of the State of New York, a former vice-president of the Mutual Life Insurance Company, and an assistant surgeon in the volunteer army during the Civil War.

